# Rheological Characteristics of Hyaluronic Acid Fillers as Viscoelastic Substances

**DOI:** 10.3390/polym16162386

**Published:** 2024-08-22

**Authors:** Gi-Woong Hong, Jovian Wan, Youngjin Park, Kathleen Chang, Lisa Kwin Wah Chan, Kar Wai Alvin Lee, Kyu-Ho Yi

**Affiliations:** 1 Samskin Plastic Surgery Clinic, Seoul 06577, Republic of Korea; cosmetic21@hanmail.net; 2 Asia Pacific Aesthetic Academy, Hong Kong; jovian.wan@apaa.org; 3 Obliv Clinic, Incheon 21998, Republic of Korea; youngjinp@gmail.com; 4 Harmony Aesthetic Clinic, Adelaide, SA 5000, Australia; harmonyaesthetica@gmail.com; 5 EverKeen Medical Centre, Hong Kong; drchan.everkeen@gmail.com (L.K.W.C.); alvin429@yahoo.com (K.W.A.L.); 6 Division in Anatomy and Developmental Biology, Department of Oral Biology, Human Identification Research Institute, BK21 FOUR Project, Yonsei University College of Dentistry, 50-1 Yonsei-ro, Seodaemun-gu, Seoul 03722, Republic of Korea; 7 Maylin Clinic (Apgujeong), Seoul 06001, Republic of Korea

**Keywords:** hyaluronic acid fillers, rheology, viscoelastic properties, elastic modulus, cross-linking agents

## Abstract

Hyaluronic acid (HA) fillers are widely used in esthetic medicine and are categorized into biphasic and monophasic types based on their manufacturing processes. To evaluate the quality of these fillers, it is essential to understand their rheological properties, which reflect their viscoelastic nature. Rheology, the study of material deformation and flow, reveals how fillers behave under stress, combining properties of solids and liquids. This study explores the fundamental principles of elasticity and viscosity, rooted in Hooke’s law of elasticity and Newton’s law of viscosity, to explain the complex behavior of viscoelastic substances like HA fillers. The distinction between biphasic and monophasic fillers lies in their chemical cross-linking processes, which impact their molecular weight, structure, and ultimately, their clinical performance. Biphasic fillers with minimal cross-linking rely on natural molecular entanglements, exhibiting lower modification efficiency and greater elasticity. Conversely, monophasic fillers, which undergo extensive chemical cross-linking, demonstrate higher modification efficiency, firmer texture, and enhanced resistance to enzymatic degradation. The study emphasizes the importance of thoroughly removing residual cross-linking agents to ensure filler safety. Understanding these rheological characteristics aids clinicians in selecting appropriate fillers based on injection sites, tissue conditions, and desired outcomes, balancing viscoelastic properties and safety for optimal esthetic results.

## 1. Introduction

Hyaluronic acid (HA) fillers, as manufactured, are generally classified into biphasic and monophasic types, each distinguished by differences arising from their specific production processes. To objectively assess the quality of each filler, we employ methods based on rheology—the study of the flow and deformation of materials—which is fundamental to understanding the characteristics of viscoelastic substances like fillers. The substances used as fillers are just one example of many viscoelastic materials encountered in everyday life, each exhibiting unique rheological properties determined by their composition, molecular weight, molecular structure, and manufacturing methods [1,2]. To fully grasp the characteristics of the fillers we use, it is crucial to understand their nature as viscoelastic substances before examining specific data metrics.

The field of rheology, or the science of deformation and flow, is relatively young, having advanced significantly only over the past few decades. Historically, the materials were assessed primarily based on their categorization as solids or liquids—studied under the principles of elasticity and fluid mechanics, respectively. However, it soon became clear that many naturally occurring substances did not fit neatly into these categories but exhibited properties of both solids and liquids. This realization led to the identification and classification of viscoelastic substances, which exhibit characteristics intermediate between solids and liquids [2].

To effectively understand viscoelastic properties, one must be familiar with the foundational principles of modern rheology, which are rooted in theories of elasticity and fluid mechanics. The theory of elasticity derives from Robert Hooke’s law, while fluid mechanics is based on Isaac Newton’s law of viscosity. Both principles, established in the 19th century, underscore the relatively recent focus on material properties in human history. It became evident that some materials display behaviors indicative of both solid-like and fluid-like properties under varying conditions. These materials, known as viscoelastic substances, have since been recognized as comprising a significant portion of the materials around us [1,2].

Thus, rheology emerged as a discipline necessary to understand substances that could not be fully explained by Hooke’s law of elasticity or Newton’s law of viscosity alone. Today, many objects used in daily life are designed based on the rheological properties studied in this field, illustrating the pervasive influence of rheology in contemporary material science.

When we think of examples of viscoelastic substances, common references include human skin, rubber balls, and springs. However, these examples are not composed of components that can flow through a tube like a fluid, and, in the authors’ view, they are not suitable comparisons to fillers. More appropriate examples of substances with characteristics similar to HA fillers are toothpaste, paint, and soft jellies, which can exhibit both solid-like and fluid-like behavior depending on the circumstances. Among these, toothpaste serves as the closest everyday analogy to the type of HA fillers we use.

Toothpaste in a tube must be forced through the tube’s opening by applying pressure, akin to how fillers are administered from a syringe through a needle or cannula into the body. Once expelled, toothpaste displays the fluid characteristics of a viscoelastic substance. On the toothbrush, it must retain a specific shape like an elastic solid; if it remained purely fluid, it would be too runny to effectively clean the teeth. Similarly, once a filler is injected, it must maintain a certain shape to enhance volume, mimicking the properties of a solid [1,2].

The rheological properties of toothpaste can vary in terms of cleaning effectiveness; some types maintain a more resistant form, offering a gritty texture, while others may spread smoothly across the brush. Some toothpastes are formulated with granular particles to enhance this solid feel, paralleling the objective of biphasic HA fillers [1,2].

This article explores the rheological data provided by manufacturers regarding HA filler products and explains the essential nature of fillers as viscoelastic substances from a rheological perspective.

## 2. Foundations of Viscoelasticity Studies

### 2.1. Elasticity

In elasticity theory, the elastic force is the force that drives an elastic object to return to its original state. This force is calculated using Hooke’s Law, typically expressed by the equation F = kΔX. Here, F represents the elastic force, k denotes the elastic modulus, which is a numerical expression of the object’s stiffness, and ΔX signifies the degree of deformation, known as the elastic limit.

The elastic modulus k is a constant determined by the material’s composition and shape. Consequently, materials with a higher elastic modulus require greater force to achieve the same degree of deformation as materials with a lower modulus, due to their inherent stiffness.

**Figure 1:**
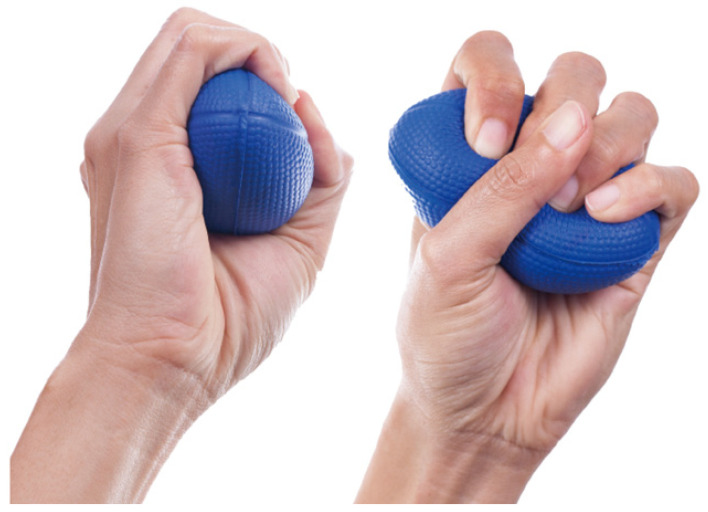
Elastic force of rubber balls increases due to a higher elastic limit compared to ordinary solid elastic materials. The figures are produced by the authors.

It is important to note that Hooke’s Law, which applies to solids, assumes similar elastic limits, meaning it is only applicable to materials with comparable structural properties at the molecular level. Common solid materials such as steel, cement, or wood, which are frequently discussed in materials science, typically have an elastic limit within 5%, indicating minimal deformation. Therefore, comparing the elasticity of human skin, rubber, or springs to these solid materials underscores the limitations of using conventional materials science methods to evaluate substances with significantly different elastic limits (Figure 1) [1,2,3].

### 2.2. Fluid Mechanics

In the field of fluid mechanics, viscosity or viscoelasticity refers to the measure of a fluid’s inherent resistance to gradual deformation by shear stress or tensile stress. Generally, a fluid with low viscosity flows easily, as it exhibits less resistance to deformation. Viscosity represents the internal friction within a fluid, which resists the layers from sliding past one another at different speeds; this behavior is governed by Newton’s law of viscosity.

Similar to how Hooke’s Law universally applies to small deformations in solids, Newton’s law of viscosity is typically valid for ordinary fluids that do not require substantial force or velocity to flow.

**Figure 2:**
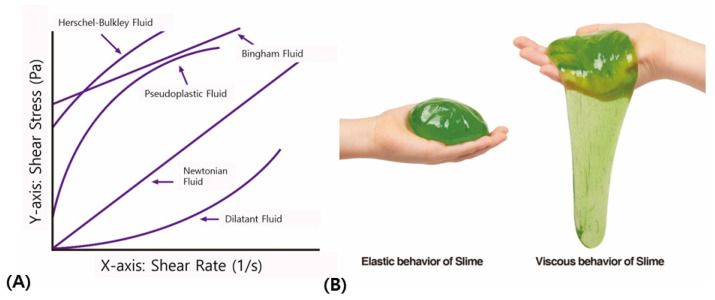
Graph depicting the contrast between Newtonian and non-Newtonian fluids in terms of fluid strain velocity (**A**). Differences in the viscoelastic behavior of slime based on its deformation history (**B**). The figures are produced by the authors.

Fluids are fundamentally classified into Newtonian and non-Newtonian fluids based on their adherence to Newton’s law of viscosity (Figure 2A). A Newtonian fluid maintains a constant viscosity regardless of the speed at which it is deformed or stressed, exhibiting a linear relationship between the velocity at which it flows and the shear stress applied. Consequently, more force is required to move higher-viscosity fluids faster. For typical fluids, the force applied to initiate flow is proportional to the resulting speed. This category includes simple liquids such as water and alcohol, which are characterized by low molecular weight solutions [1,2,4].

Non-Newtonian fluids, also referred to as plastic fluids, include colloidal high-concentration solutions that exhibit a robust three-dimensional network among particles, requiring a certain threshold of stress to initiate flow. In these fluids, the relationship between flow speed and stress is non-proportional, as flow commences only after applying a sufficient level of stress that surpasses a critical threshold. Typically, discussions of fluid viscosity concentrate solely on the overall resistance to flow resulting from internal and external friction, without considering the internal particle structure. However, in the case of polymeric compounds, the specific structures formed between components require substantial force to disrupt. It is proposed that this structural viscosity affects cohesion—the force that enables particles within a material to return to their original configuration after being disturbed. This structural viscosity suggests that a significant initial force is necessary to disrupt the material’s structure and initiate movement. Once the structural viscosity is overcome, the material can flow with considerably less force [1,2,4].

### 2.3. Rheology

Rheology is the study of the flow of matter, concentrating on how materials respond to deformation, whether exhibiting elastic solid-like or viscous fluid-like behavior. Unlike solids, where elastic theory applies and deformation is solely defined by strain within elastic limits, rheology addresses materials whose stress response depends on the rate at which they are deformed or their rate of strain.

Materials commonly encountered in everyday life, such as leather, rubber, and polymers, display both viscous and elastic characteristics. Consequently, a single material may behave differently under varying conditions: it may resist deformation like an elastic body depending on the extent of strain or resist the rate of deformation like a viscous fluid. The dominance of either elasticity or viscosity in a viscoelastic material largely depends on external conditions, such as the manner in which the material is deformed, making stress dependent on the deformation history. Thus, stress in viscoelastic materials cannot be simplified into basic equations.

A familiar example of viscoelastic behavior can be observed in a simple school science experiment, i.e., mixing cornstarch and water to form a non-Newtonian fluid. When forcefully squeezed, this mixture behaves like a solid, forming a lump; however, when left undisturbed, it spreads and flows like a liquid.

Similarly, a child’s toy like slime (also known as Silly Putty or Flubber), a polymer mixture, serves as a useful illustration of viscoelastic properties akin to those seen in HA fillers. Slime can be molded and shaped like clay; when rolled into a ball and thrown to the ground, it bounces back like a rubber ball. Conversely, if placed on a perforated surface, it will slowly flow and drip through the holes over time [1,2,4].

In the former scenario, rapid deformation of the slime results in an elastic response, while in the latter, the slow, gravity-driven deformation showcases its viscous fluid characteristics. This dependency of a viscoelastic material’s response on its deformation history is what defines viscoelastic behavior (Figure 2B).

Viscoelastic materials have higher strain limits compared to typical solids and form strong three-dimensional structures not seen in ordinary fluids, resulting in what is referred to as structural viscosity rather than mere fluid viscosity.

The physical laws that describe the relationships between applied deformations and the resultant stresses in materials are known as constitutive equations. While Hooke’s Law and Newton’s law of viscosity represent simpler forms of these equations, rheology seeks to develop and evaluate more complex constitutive equations that describe the viscoelastic and flow behaviors of materials with intricate compositions. Thus, rheology can be defined as a branch of mechanics of materials focused on the study of materials whose properties cannot be adequately described by traditional elasticity or simple fluid viscosity models.

## 3. Rheological Indicators Used for Evaluating Viscoelastic Hyaluronic Acid Fillers

HA fillers are fundamentally viscoelastic materials, and their properties are assessed using common rheological methods within the field of rheology, which studies the viscoelastic behavior of materials. These evaluations involve measuring various rheological properties in response to different types of forces applied to the fillers, including compression, torsion, stretching, and lateral shear.

In the laboratory, a rheometer is typically employed to apply these forces to the HA fillers [1,2,4]. By employing attachments such as parallel plates, cone and plate systems, and concentric cylinders, the rheometer applies the four types of forces to assess how the fillers deform and recover.

Rheology frequently measures the dynamic viscosity of materials by subjecting them to electrical vibrations, which act as electrical shearing forces. The magnitude of these vibrations can be varied, with the rate at which they increase referred to as the electrical shearing rate. As the shearing rate increases, viscoelastic materials adapt by rearranging their internal structure in response to the vibrations. Depending on their viscoelastic properties, materials exhibit varying responses; those with higher viscosity exhibit increased stress responses as the shearing force increases, slightly increasing their elasticity. Conversely, these vibrations decrease the viscosity as the structural viscosity of the material breaks down.

For materials such as a free HA solution, which primarily display fluid characteristics, the structural cohesion is weak, making them responsive to electrical vibrations and thus altering their viscoelastic properties.

**Figure 3:**
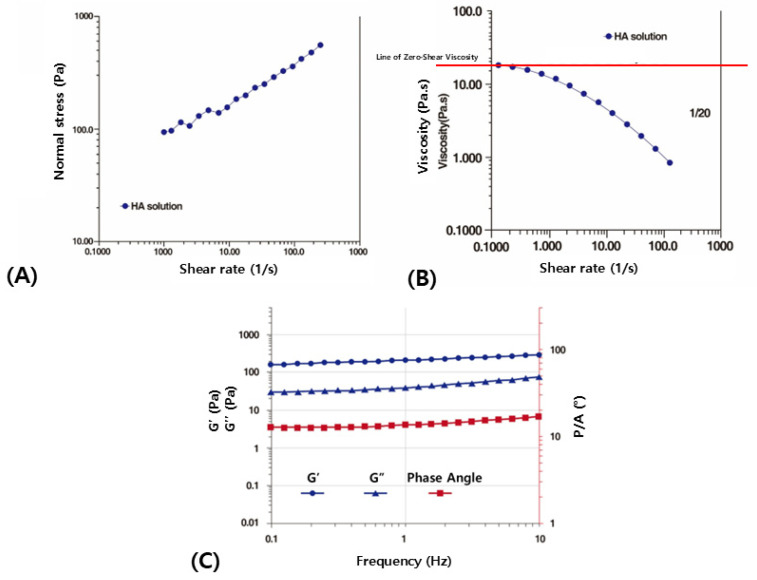
Normal stress growth with increasing shear rate in the free HA solution (**A**) (standard deviation of the normal stress is 105.37 Pa). Change in zero-shear viscosity with increasing shear rate in the free HA solution (**B**) (standard deviation of the viscosity is 4.2 Pa·S). Stabilized rheological pattern of cross-linked HA filler with increasing shear rate (**C**). The concentration of the hyaluronic acid was 20 mg/mL. The figures are produced by the authors.

Typically, electrical vibrations between 0.1 and 10 Hz are applied. As the frequency of the vibration increases, the normal stress generated in response increases the elastic modulus (Figure 3A), while the viscous modulus decreases as the structural viscosity diminishes (Figure 3B).

This phenomenon is analogous to making Dalgona coffee, where continuously stirring a mixture of coffee, sugar, and water changes its properties from a fluid state to a viscous state. While this might appear as a simple effect of stirring, from a rheological perspective, the act of stirring introduces a shearing force that rearranges the molecular structure of the mixture, thereby altering its physical properties (Figure 3C).

When HA fillers are subjected to external forces, they display both elastic and viscous behaviors. If subjected to forces within their elastic limit, they return to their original state once the forces are removed. However, if the forces exceed the elastic limit, the structure breaks down and does not return to its original state, although some form of reconstitution might occur due to the electrical attractions among the filler particles. This reconstitution is often referred to as cohesion.

**Figure 4:**
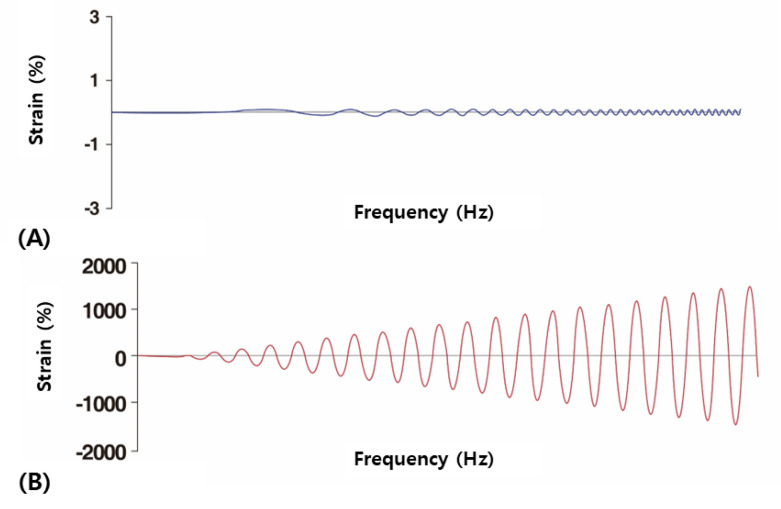
Linear frequency sweep test where the amount of deformation (strain) remains constant while the frequency increases (**A**). Amplitude sweep test where the amount of deformation (strain) increases (**B**). The figures are produced by the authors.

Rheological testing using oscillatory rotation with parallel plates in a rheometer typically takes place at room temperature with a frequency range of 0.1 to 10 Hz and is known as a linear frequency sweep test (Figure 4A,B).

The viscoelastic nature of HA fillers means that their behavior under stress can vary. When applied slowly, such as through a syringe, their viscous properties facilitate easy passage through the needle or cannula. However, if pushed rapidly, the filler may resist movement due to its elastic properties, which react more significantly under sudden stress.

In clinical settings, understanding the rheological properties of HA fillers aids physicians in selecting the appropriate filler for their procedures to achieve optimal results. Thus, rheological indicators not only provide a means to quantitatively measure material properties but also offer insights into their clinical implications, enhancing their application in medical esthetics [5].

When considering the viscoelastic properties and safety of HA fillers, several factors must be taken into consideration, including the specific application area (e.g., superficial versus deep injections), the desired duration of effect, and the patient’s individual skin characteristics. The viscoelastic properties, particularly the storage modulus G′, are crucial in determining the filler’s ability to maintain shape and resist deformation under stress. This is essential for achieving natural-looking results and ensuring the longevity of the filler. An optimal range for the storage modulus G′ typically lies between 100 and 500 Pa for facial applications. This range provides sufficient firmness and elasticity to offer effective tissue support while minimizing the risk of adverse events such as nodule formation or migration.

### 3.1. G′: Elastic Modulus

The elastic modulus, denoted as G′, measures the degree of elasticity of a filler, indicating its ability to resist deformation from external forces and maintain its original shape. Unlike hard solids, the deformation limits—referred to as strain—vary among typical elastic materials. Consequently, the same force applied to different materials results in varying degrees of deformation, depending on the stiffness represented by the elastic modulus and the extent of strain each material can undergo.

Typically, the elastic modulus is measured in Pascals (Pa) at 1 Hz with a 1.0 mm gap, as indicated by the Malvern Kinexus, a rotational rheometer manufactured by Malvern. One Pascal represents the pressure exerted when one Newton of force is applied over an area of one square meter.

The notation ‘1 Hz-gap 1.0 mm’ refers to the application of electrical vibrations using parallel plates in oscillatory rotation, with the vibration frequency increased by 1 Hz per 1 mm gap. This setting measures the filler’s elasticity as it responds under these conditions.

In practice, materials with a higher G′ value require more force to achieve the same level of deformation compared to those with a lower G′. This characteristic implies that fillers with higher G′ values are relatively stiffer and can better maintain their shape under external pressure. The term ‘storage modulus’ is often used interchangeably with elastic modulus, referring to the energy stored in a material during deformation, which is later released as the material returns to its original shape once external forces are removed [5,6].

When the same external force is applied, the degree of deformation will vary among fillers depending on their strain characteristics. However, achieving a similar level of deformation across different fillers will require more force for those with greater G′ values. Consequently, fillers with higher G′ values are better equipped to withstand external forces or pressures post-injection, thereby maintaining their structural integrity and shape [5,6,7,8].

**Figure 5:**
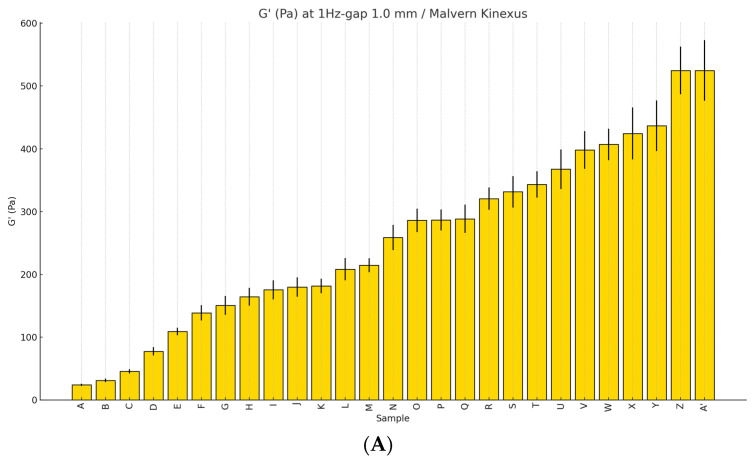

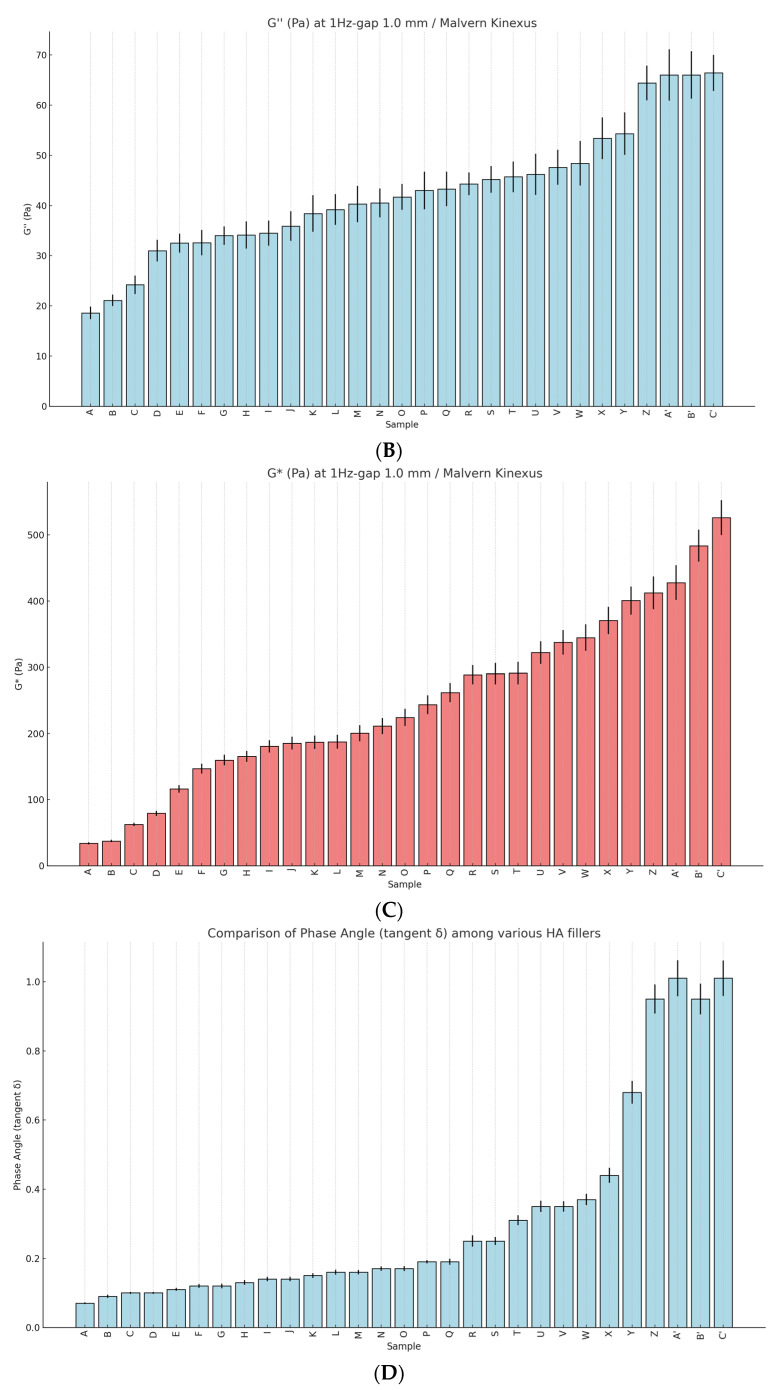
Comparison of the elastic modulus of various HA fillers (**A**). Comparison of the viscous modulus of various HA fillers (**B**). Comparison of the complex modulus of various HA fillers (**C**). Comparison of phase angle (tangent δ) among various HA fillers (**D**). The standard deviations were calculated based on the standard formula for standard deviation, which is the square root of the variance. For each dataset, we first calculated the mean, followed by the variance (the average of the squared differences from the mean), and then took the square root of the variance to obtain the standard deviation. The figures are produced by the authors.

The following chart (Figure 5A) organizes several commonly used HA fillers in domestic markets according to their elastic modulus, arranged from the lowest to the highest values. This graph highlights the broad spectrum of elasticities among different fillers.

It is important to recognize that the numerical values of elasticity are not absolute but can vary depending on experimental conditions and the specific rheometer used. Nevertheless, under consistent conditions, fillers with superior elasticity will typically exhibit higher G′ values. Consequently, these elasticity measurements can assist in selecting the most suitable filler based on the intended application and performance criteria.

### 3.2. G″: Viscous Modulus

The viscous modulus, denoted as G″, quantifies the viscosity or the sticky characteristic of a filler. Unlike elastic bodies, viscous fluids continue to deform under external force and do not return to their original shape once the force is removed. In such fluids, the shape continuously changes under external force, and the energy applied is not restored to the original form but is instead dissipated as lost energy, hence the term “loss modulus” [5,6,7,8,9,10].

Materials with high viscosity possess molecular structures that are sticky and tightly bound, requiring more significant force to sustain continuous deformation compared to less viscous, more fluid substances. Consequently, a filler with a higher G″ value indicates high viscosity among the filler particles, making it challenging to deform and move smoothly within a syringe during injection. However, once injected, such fillers maintain their shape better under external forces or movement due to their resistance to deformation.

In monophasic HA fillers engineered to have high viscosity, the filler particles themselves are sticky and clump together, maintaining their form robustly against external forces and displaying high elastic properties similar to those of biphasic fillers [10].

Biphasic fillers, due to their manufacturing characteristics, are typically firm with good elasticity but are expected to have lower viscosity. However, laboratory measurements of viscosity often show that even biphasic fillers with high elasticity also display high viscous values. It is crucial to understand that the absolute numbers of viscosity are less important than the ratio of elasticity to viscosity. While biphasic fillers may show high viscous measurements, this is not due to the inherently sticky nature of the materials but rather because the laboratory tests measure viscosity in firm, viscoelastic substances. Therefore, the phase angle value, which represents the ratio of viscous modulus to elastic modulus, is another crucial metric to consider alongside measurements of both elasticity and viscosity.

Despite their high viscosity values, biphasic fillers should not be regarded as having superior viscous properties solely based on their high numeric values. The high elasticity-to-viscosity ratio indicates that the actual viscous nature is reduced compared to monophasic fillers [5,6,7,8,9,10,11].

The following chart (Figure 5B) presents the viscous values of various HA fillers used in South Korea, ordered from the lowest to the highest viscosity values. Monophasic fillers are generally arranged in order of increasing viscosity, correlating with their elasticity. However, despite having higher absolute viscous values, biphasic fillers should not be deemed to have better viscosity compared to monophasic fillers with lower values. Understanding these manufacturing characteristics and the viscous value of each filler is essential for selecting the appropriate filler for specific treatment goals, just as with elasticity measurements.

### 3.3. G*: Complex Modulus

The complex modulus (G*) is a metric that integrates both the elastic modulus (G′) and the viscous modulus (G″). G′ quantifies the extent to which an elastic material resists deformation under external forces and returns to its original shape, while the G″ measures the degree to which a viscous fluid continues to deform under such forces. Rather than evaluating these properties separately, the complex modulus provides a unified measure of a material’s overall response to deformation.

For viscoelastic substances like fillers, which exhibit characteristics of elastic solids and viscous fluids, the complex modulus is particularly valuable. It encapsulates the combined effects of a filler’s elasticity and viscosity, enabling a more straightforward prediction of how the filler will respond to external forces without the need to separately assess its individual elastic and viscous properties.

In monophasic fillers, which typically display high viscosity due to their manufacturing process, elasticity often correlates proportionally with this high viscosity, leading to a high complex modulus. This correlation allows for a more intuitive inference of the viscoelastic behavior of these fillers. However, for biphasic fillers, a high absolute value of the complex modulus should not be interpreted as an indication that both elasticity and viscosity are equally high.

The chart below (Figure 5C) presents the complex moduli of various HA fillers used in South Korea, calculated from their respective elasticity and viscosity values, and arranged in ascending order. This arrangement assists practitioners in assessing the overall mechanical properties of the fillers and selecting the most suitable filler based on its combined elastic and viscous characteristics.

### 3.4. Phase Angle (Tangent δ)

The phase angle, denoted as tangent δ, serves as an indicator to estimate whether the properties of an HA filler resemble those of an elastic solid or a viscous fluid. This measurement is calculated as the ratio of the viscous modulus (G″) to the elastic modulus (G′) under consistent conditions for each filler, expressed as G″/G′.

A phase angle value approaching 1 suggests that the filler’s viscous properties predominate over its elastic properties, indicating that the filler behaves more like a viscous fluid than an elastic solid. Conversely, a phase angle value less than 1 signifies that the filler’s elastic properties are more pronounced relative to its viscosity. Fillers with a lower phase angle are thus more similar to traditional elastic solids, exhibiting stiffer characteristics.

As the phase angle nears 0, it implies that the filler exhibits minimal viscous behavior and is closer to a pure elastic solid, with almost no characteristics typical of viscous fluids.

The chart below (Figure 5D) organizes popular domestically available HA fillers by their calculated phase angle values, ranging from those closest to 0 (indicating stiffer, more elastic fillers) to those closer to 1 (indicating softer, more viscous fillers).

Among monophasic fillers with similar elastic values, the phase angle can still vary significantly due to different manufacturing conditions [11,12,13,14]. These variations in phase angle not only reflect differences in viscous properties among monophasic fillers but also influence the degree of cohesiveness—a characteristic commonly associated with viscous fluids. Further discussion on the impact of phase angle on cohesiveness among monophasic fillers will follow.

### 3.5. Cohesion

Cohesion refers to the attractive force between molecules within a substance, which causes molecules to pull together. This force significantly influences the behavior of both solids and liquids. For instance, molecular cohesion accounts for phenomena such as gases condensing into liquids or liquids solidifying into solids.

When liquids like water or mercury are spilled, they form droplets rather than spreading out flat due to cohesion among their molecules. Similarly, viscous fluids naturally tend to clump together because of this cohesive force. Cohesion should not be confused with adhesion, which is the force acting between different materials. While cohesion pertains to the attraction within the same substance, adhesion describes the attraction between different substances [14].

For example, water tends to spread on a glass surface due to the adhesive forces between the water molecules and the glass, demonstrating a moderate level of adhesion. In contrast, water forms droplets on a wax-coated glass surface because the adhesive forces are weaker compared to the cohesive forces within the water.

On a flat surface, water droplets maintain a rounded shape. However, on slanted surfaces, such as a leaf, droplets spread out and flatten because the adhesion between the water and the leaf is stronger than the cohesion within the water, preventing the droplets from rolling off. Mercury, known for its strong cohesive forces, retains its rounded shape even on tilted surfaces due to its dominant cohesive forces over any adhesive forces with the surface.

In the context of HA fillers, some manufacturers emphasize their products’ ability to adhere to vertical surfaces without dripping, highlighting strong adhesion rather than cohesion. This distinction is important, as the primary factor in such demonstrations is adhesion, not cohesion.

Viscous fluids typically do not spread out but form cohesive droplets, and viscoelastic fillers exhibit this property due to their viscosity. In HA fillers, particularly monophasic ones, the cohesive forces are stronger because the filler particles are more tightly bound by the cohesive forces inherent in their molecular structure [8,15].

Cohesion is a critical property for fillers injected into the human body, as external factors can alter the shape of the filler post-injection. Understanding the level of cohesion between filler particles is essential for predicting how well the filler will return to its intended shape after deformation [8,15,16,17,18,19].

A high degree of cohesion in a filler indicates that the filler particles can better withstand external forces and maintain their intended form. Conversely, fillers with weaker cohesion may not recover their shape as effectively after deformation. This property is increasingly considered when selecting a filler for a specific treatment area, the intended effect, and the patient’s skin and tissue characteristics [20]. Thus, understanding and measuring cohesion among filler particles is becoming an important aspect of choosing the right filler for optimal results.

Cohesion, as opposed to the cross-linking seen in some HA fillers, arises from the natural electromagnetic attraction among molecules rather than from rigid molecular bonding. This means that cohesion can be easily disrupted by external stimuli, unlike elasticity, which robustly resists deformation [7].

Therefore, a high level of cohesion does not necessarily imply that a filler will maintain its shape effectively against external forces. For example, areas such as the nasolabial folds or the tip of the chin, where strong ligamentous tissues and firm skin continuously exert pressure, may not be ideal for fillers that rely heavily on cohesion. Such fillers are better suited for areas like the cheeks, where they can adapt smoothly to minor everyday movements and pressures, returning to their original form once the external force is removed [7,20].

**Figure 6:**
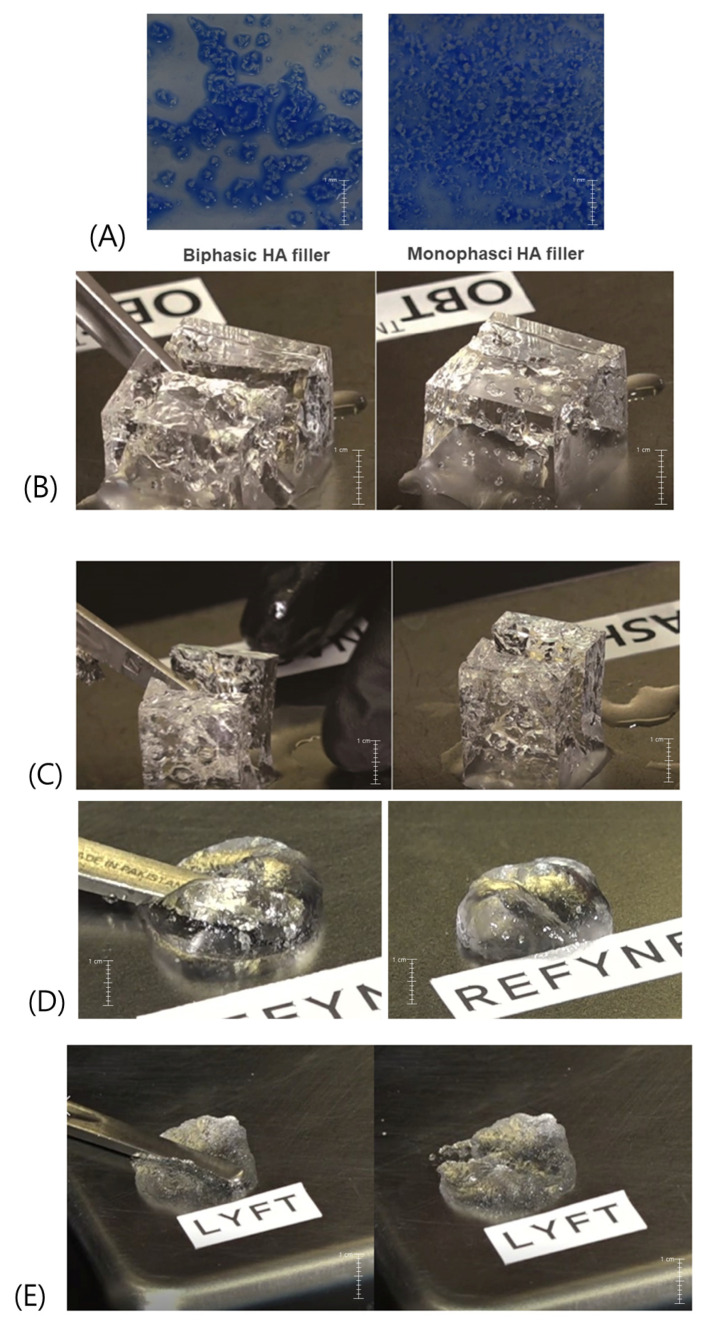
Comparison of particle aggregation and distance between particles in biphasic and monophasic HA fillers. When saline is injected into the product to dilute it and shaken, the filler particles separate from each other. Biphasic fillers, despite having good elasticity, have low viscosity, causing the particles to aggregate and spread out due to weak cohesiveness. On the other hand, monophasic fillers have strong cohesiveness based on their viscosity, resulting in the particles remaining together to some extent without dispersing (**A**). Test for structural restoration ability by cohesion in monophasic HA gel mass (**B**). Test for the ability of structural restoration by cohesion in biphasic HA gel mass (**C**). Test for structural restoration ability by cohesion in monophasic HA gel product (**D**). Test for structural restoration ability by cohesion in biphasic HA gel product (**E**). The figures are produced by the authors.

To understand the differences in cohesion between biphasic and monophasic HA fillers, experiments were conducted on the gel mass and the actual particles of the product before final formation (Figure 6A). It was observed that the biphasic gel mass, with its stronger elasticity, requires significantly more force to deform compared to the monophasic gel mass. Although the monophasic gel mass deforms more readily with less force, it demonstrates a higher degree of deformation before breaking. This indicates that exceeding the elastic limit can destroy the structure of both gel types, regardless of their elasticity.

When a gel mass cube breaks, a truly elastic substance would not be able to restore its original structure after the removal of the external force. However, because HA fillers are viscoelastic, the cohesive forces within the viscous fluid enable the HA molecules to reassemble and repair the broken structure. In the case of the monophasic gel mass, which exhibits superior cohesion, the HA molecules can reattach more effectively, filling in the gaps created by the breakage. Consequently, after repair, the traces of the breakage are barely noticeable due to the strong cohesive bonding among the molecules (Figure 6B). This highlights the significant role of cohesion in determining how well a filler recovers after deformation, making it a crucial property to consider when selecting the most suitable filler for a particular application.

In contrast, biphasic HA fillers, which inherently possess the properties of viscous fluids, exhibit only moderate aggregation of HA molecules around breaks, unlike the more pronounced cohesion observed in monophasic gel masses. Consequently, the gaps are not fully filled, leaving visible signs of fracture (Figure 6C).

The difference in cohesion between biphasic and monophasic HA fillers can be observed directly in their product forms. For instance, tests conducted with 1 mL each of biphasic and monophasic HA fillers from Galderma involved extruding the fillers from syringes, applying pressure with a stick, and then observing the changes after the pressure was released. When excessive force was applied, the HA gel mass in cube form exhibited cracking, whereas the filler products demonstrated flattening deformation. Upon removal of the applied force, the robust cubic gel mass showed HA molecules clustering to repair the cracks, whereas the filler products rounded up like water or mercury, indicating significant shape recovery due to superior cohesion. In particular, monophasic HA fillers displayed deformation with minimal applied force, but the area previously compressed partly regained its shape after the force was removed, attributed to their high cohesion (Figure 6D).

Conversely, biphasic HA fillers, characterized by strong physical cross-linking among HA molecules, exhibit good resistance to external forces due to their elasticity but display weak cohesion among particles. Consequently, when subjected to forces exceeding their elastic limit and then released, these fillers do not recover their original shape effectively because the weak cohesion fails to reunite the particles, leaving them in a deformed state (Figure 6E).

The clinical significance of cohesion testing results is that they guide the selection of fillers based on observed properties. Fillers with high cohesion perform well in areas with minimal external forces, maintaining their shape even after significant deformation beyond their elastic limit. However, high cohesion alone does not universally qualify a filler as superior. In areas with thick, dense skin and tissue, continuous external pressure will deform the filler, keeping it compressed. Therefore, fillers with high elasticity are essential in areas like the masseter or chin where enduring external pressure is crucial. Fillers with low elasticity but high cohesion may spread under the skin in these areas, failing to maintain a satisfactory shape.

Additionally, it is important to consider the nature of viscoelastic bodies, which alter their properties based on deformation history. When a suitably viscoelastic filler is gently tapped under soft skin, it resists deformation and feels firm. However, if the same area is slowly pressed, the filler exhibits more viscous properties and compresses further; upon pressure release, it gradually returns to its original volume. Fillers with only high cohesion may feel squishy and fail to rebound effectively, potentially absorbing water and appearing swollen [21,22,23,24].

These factors are crucial for predicting how a filler will perform post-injection, particularly regarding its resistance to deformation and its ability to return to its original state (Table 1).

The influence of cohesion in HA fillers is primarily determined by the molecular characteristics and concentration of HA. Generally, a higher concentration and larger molecular weight lead to stronger cohesive forces among molecules [1,6]. Additionally, the manufacturing process impacts cohesion; for example, monophasic fillers typically exhibit greater cohesion compared to biphasic ones due to variations in viscosity introduced during production. Proper hydration during manufacturing ensures sufficient hydrogen molecules are available to form stable hydrogen bonds between particles, thereby enhancing cohesion. Furthermore, the size and uniformity of filler particles affect cohesion; particles that are more uniform and appropriately sized tend to show stronger cohesion. The phase angle (tangent δ), which indicates the ratio of viscosity to elasticity, also plays a role in cohesion [25,26,27,28]. However, a high phase angle does not necessarily imply better cohesion. While a higher phase angle suggests improved cohesive properties under similar elasticity conditions, values exceeding 0.25 may result in overly viscous characteristics that compromise the structural integrity required to maintain shape, making cohesion less effective. This phenomenon is evident in fillers that are extensively diluted with free HA solution for ease of application, which often exhibit properties unsuitable for volume retention due to high fluidity and significantly reduced elasticity.

Currently, the selection of fillers considers the required viscoelastic and cohesive properties suitable for the intended treatment area. However, cohesion is not universally essential across all viscoelastic materials, presenting challenges in standardizing measurement techniques in rheology. As a result, selecting fillers becomes complex in the absence of established methods to reliably assess cohesion. This discussion will later explore various methods for inferring cohesion, emphasizing empirically valid approaches to assist in product selection.

## 4. Methods for Measuring Cohesion

### 4.1. Perceived Cohesion Test

This method involves manually assessing the stickiness of filler to gauge its cohesiveness. In this test, the degree of tackiness experienced when touching the filler indicates the strength of the cohesive forces holding the particles together. However, it is important to note that this test measures perceived stickiness rather than providing an objective numerical value for cohesion. The tackiness felt on the hands may reflect adhesive forces as much as, or more than, purely cohesive forces.

**Figure 7:**
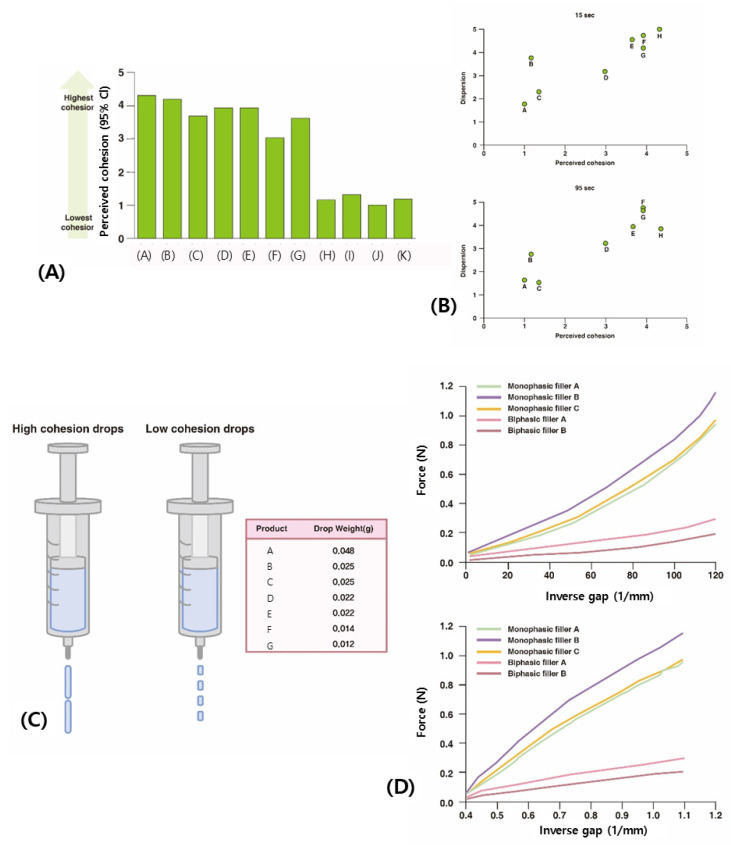
Perceived cohesion test (**A**). Dispersion test (**B**). Drop weight test (**C**). Compression force test (**D**). The figures are produced by the authors.

The procedure requires medical professionals to handle various types of fillers and then rate the level of stickiness to the skin, the extent to which the filler particles cling to each other, and the molecular force resisting separation of the particles. Ratings are assigned on a scale from 1 to 5, with a product consistently ranking closer to 5 inferred to have higher cohesion (Figure 7A). This subjective method offers a comparative reference rather than precise, quantifiable data on cohesion [25,26,27,28].

### 4.2. Dispersion Test

This test evaluates the dispersion of 1 mL of HA filler, dyed with toluidine blue, in water. It assumes that fillers that disperse easily in water are likely to exhibit weaker cohesion, indicating that the particles do not cluster effectively to form a stable structure.

The degree of dispersion is initially rated on a scale from 1 to 5: 1 indicates full dispersion, 2 indicates partial dispersion, 3 indicates partial dispersion/partial cohesion, 4 indicates partial cohesion, and 5 indicates full cohesion. This rating reflects how much the filler particles spread out in water, ranging from complete scattering to remaining fully clustered.

The dyed HA filler is observed at various time intervals—15 s, 70 s, 95 s, 5 min, and 10 min after immersion in water—to assess how the particles disperse. During the first 95 s, biphasic HA fillers typically show significant dispersion due to weaker inter-particle bonds, whereas monophasic HA fillers exhibit somewhat greater resistance to dispersion. The dispersion grade of each filler over time provides insight into its cohesive properties.

A graph correlating perceived cohesion ratings with dispersion test results indicates that products with higher cohesion ratings tend to maintain particle integrity better in the initial stages (Figure 7B). However, by 5 min, as hydration progresses, even monophasic HA fillers begin to disperse, and by 10 min, they are fully dispersed.

This test illustrates that initial cohesion can be estimated based on how well the particles remain bonded in water. However, measuring differences becomes challenging over time due to the hydration process. Furthermore, while a high dispersion test score may suggest strong cohesion, it is important to recognize that in products with fluidity exceeding 0.25, even if particles exhibit strong cohesion, the practical significance of such cohesion diminishes if the product’s inherent elasticity is low, making it more similar to a viscous fluid than a solid-forming material.

### 4.3. Drop Weight Test

This test assesses the cohesive properties of HA fillers by measuring the length and continuity of filler strands as they are extruded through the opening of a syringe and allowed to fall under gravity. Stronger inter-particle adhesion results in longer and heavier strands, indicating higher structural viscosity and cohesion (Figure 7C). Typically, biphasic fillers exhibit weaker cohesion, which leads to the formation of smaller, lighter drops as the connections between particles break more readily compared to monophasic fillers. However, this test is subject to variability due to factors such as syringe capacity and nozzle size. Additionally, if the filler tends to adhere to the syringe rather than falling, it may reflect adhesive forces, which could influence the interpretation of cohesion.

### 4.4. Compression Force Test

This test evaluates the cohesive properties of HA fillers by applying a force that exceeds the elastic limit, breaking down their elastic properties, and then allowing time for the structure to potentially recover through the cohesive forces among HA molecules. After this period, a minor deformation is introduced, and the resulting “inverse gap” between the particles is measured. A smaller inverse gap indicates that the particles remain closer together, suggesting stronger cohesion.

When HA fillers are compressed beyond their elastic limits, the structure breaks down, increasing the distance between particles. If the applied force is then removed, biphasic HA fillers typically do not reassemble effectively due to their weaker cohesion, which is characteristic of their viscous, fluid-like properties. Consequently, even after a recovery period, biphasic fillers exhibit significant particle separation, which increases when minor forces are subsequently applied. This behavior demonstrates their diminished ability to resist external forces due to a loss of elasticity and poor cohesive recovery (Figure 7D).

In contrast, monophasic HA fillers, with their relatively lower elastic modulus, initially deform with less force compared to biphasic fillers, leading to structural breakdown. However, once the force exceeds the elastic limit and disrupts the structure, the inherent viscous fluid-like cohesion of monophasic fillers causes the particles to gather and narrow the inter-particle gaps, partially restoring the deformed structure. After allowing a recovery period, when a minor force is applied, monophasic fillers, despite reduced elasticity, maintain particle cohesion and resist further separation under minor stress, thus preserving the filler structure.

This experiment, conducted by Allergan, was designed to highlight the advantages of Juvederm, a monophasic HA filler, compared to Restylane NASHA, a biphasic HA filler produced by Galderma. Allergan first applied significant force to disrupt the elasticity of the fillers, allowed time for structural recovery through cohesion, and then measured the extent of particle separation under a smaller applied force. A smaller increase in distance with applied force indicated greater cohesion, and products were ranked accordingly. Allergan termed this property “cohesivity”, although it is not formally recognized in rheology. They proposed a formula where a filler’s ability to maintain shape, termed “lifting capacity”, equals G′ (gel firmness and particle size) multiplied by cohesivity. According to Allergan, both the gel’s firmness (G′) and its cohesion (cohesivity) impact the filler’s ability to withstand external stimuli and maintain shape [25,26,27,28].

This formula may be valid in certain contexts but is not universally applicable to all HA filler treatments. While G′ represents the capacity to resist deformation and maintain shape under external pressure, “cohesivity”, as measured in Allergan’s experiment, reflects the ability to regain form after mild external forces have disrupted the filler’s structure. Thus, it is more relevant to areas requiring minimal resistance to deformation, where the filler must recover its shape post-stimulation rather than withstand strong external forces. The compression force test is designed to evaluate this recovery capacity rather than the ability to endure significant deformation.

In Allergan’s tests, the process involved initially flattening the filler under considerable force, allowing approximately 120 s for the filler particles to coalesce due to cohesion, and then applying a minor force to measure the extent of particle movement. The force was gradually increased from zero to about 1.2 N, a force much smaller than what is typically needed to disrupt filler elasticity, as shown by the graph’s vertical axis measurements. Although the force applied was minimal, the gradual increase over time revealed significant differences in the behavior of different filler types. For example, to widen the inverse gap to 1.2 mm, the biphasic HA filler Restylane NASHA required only 0.2 N, while the monophasic HA filler Juvederm needed up to 1.2 N, indicating greater resistance to compression force by Juvederm.

It is important to note that the force magnitude used in these tests, where 1 N corresponds to the force needed to lift approximately 0.1 kg at the Earth’s surface, is relatively small—similar to the force required to press a computer keyboard key. Thus, the concept of “lifting capacity” proposed by Allergan is accurate for areas subjected to light external forces, where fillers need to recover shape after minor deformations. However, if the term is intended to describe the ability of fillers to support dense, strong tissues against significant pressure and maintain volume based on elasticity, then the term may be less appropriate. Maintaining form under such conditions would require greater resilience and elasticity than what Allergan’s formulation of lifting capacity, based solely on gel firmness and cohesivity, may suggest.

### 4.5. Creep Deformation and Recovery Test

The Creep Deformation and Recovery Test evaluates a filler’s ability to regain its original shape after being subjected to compressive or distortive forces, based on its structural viscosity and the cohesive electrostatic forces among its particles. This test is considered clinically relevant for assessing how well a filler maintains its fundamental structure after deformation.

Creep deformation refers to the progressive deformation of a material under a constant load over time. For instance, when a constant weight is suspended from a material, it stretches due to the applied load. If the material is elastic, it will deform within its elastic limit under the weight and maintain this deformation as long as the load is applied. Once the weight is removed, the material’s elasticity allows it to return to its original shape. However, if the weight exceeds the material’s elastic limit, the material’s structure may break, preventing it from returning to its original shape after the load is removed, as it remains in a deformed, damaged state. In contrast, a viscous fluid under the same conditions would continue to flow or deform at a constant rate of strain.

Viscoelastic materials, which possess both elastic and viscous properties, exhibit behaviors characteristic of both solids and fluids in a creep deformation test. Initially, they respond elastically to the load with immediate deformation. If the load is within their elastic limit, the deformation ceases at a certain point. However, if the load exceeds the elastic limit, the viscoelastic material will continue to stretch slowly over time due to its viscous properties. This dual response allows viscoelastic materials to adapt to both immediate and prolonged stresses, demonstrating a unique combination of shape retention and flow.

To simulate conditions similar to those experienced by fillers under regular stress and gradual deformation, viscoelastic materials are subjected to loads heavier than they can nominally support. The process involves applying a sustained load that exceeds the material’s elastic limit, inducing both immediate elastic deformation and slower, ongoing deformation. After a specified period, the load is removed to assess the material’s ability to recover.

For biphasic fillers, which exhibit stronger viscoelastic solid properties, the recovery driven by interparticle cohesion is generally weak, resulting in minimal shape recovery. Conversely, monophasic fillers, characterized by stronger viscous properties and better particle cohesion, can more effectively reverse deformations caused by creep—the gradual deformation that continues until the load is removed. This slow return to the original form is termed creep recovery.

**Figure 8:**
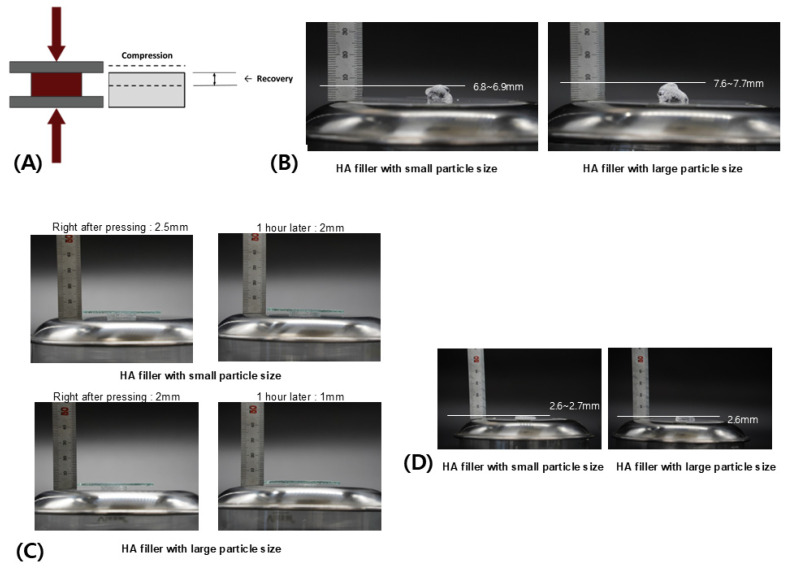
Creep deformation and recovery test (**A**). Comparison of shape and size of HA filler gel with different particle sizes when there is no external deformation (**B**). Comparison of response to creep deformation between HA filler gels with different particle sizes (**C**). Comparison of creep recovery of HA filler gel with different particle sizes (**D**). The figures are produced by the authors.

Thus, the Creep Deformation and Recovery Test measures the extent of deformation under a continuous load beyond the elastic limit and the subsequent recovery after the load is removed. This test evaluates how a viscoelastic material initially responds to stress with immediate elastic changes and how it slowly recovers from further deformations over time, showcasing both its immediate and delayed response capabilities (Figure 8A).

When viscoelastic materials with a viscous texture, such as certain monophasic fillers, undergo slow creep deformation, they typically demonstrate gradual recovery characteristics. This recovery is largely driven by the inherent cohesion among the material’s molecules—an intrinsic bonding force within the substance. Even among similar monophasic fillers, variations in cohesion can arise due to differences in fluidity, particle size, and consistency, as previously discussed [22,24,29].

To illustrate this, an experiment compared the extent of creep deformation and recovery between two monophasic fillers with different particle sizes but similar viscoelastic properties. Initially, equal volumes of each filler were extruded from syringes onto a flat surface to compare their heights without any load. The filler with larger particles exhibited a higher initial height due to greater spacing between particles.

Upon applying a weight that exceeded the elastic limit, the degree of compression and reduction in space between particles should be similar if the viscoelastic properties are consistent. However, even if the rate of reduction is the same, fillers with larger particles experience a greater absolute reduction in length due to the larger individual particle area and spacing. This results in a significantly lower height after initial elastic deformation. The subsequent slow progression of creep deformation also appears more pronounced in these larger-particle fillers due to the greater ratio of space between particles (Figure 8B).

After the load is removed, the degree of shape recovery facilitated by particle cohesion was assessed. Fillers with smaller particles, which inherently have less space between them, demonstrated superior cohesion. This allowed them to reassemble more effectively and recover height. Consequently, when comparing the original height with the height after deformation and subsequent recovery, smaller-particle fillers exhibited more effective creep recovery than their larger-particle counterparts (Figure 8C).

In an alternative approach, centrifugal force generated by rotation was applied to measure how well the spacing between particles was maintained without outward dispersion. Under this force, fillers with smaller particle sizes exhibited better cohesion, resulting in less dispersion of the filler material (Figure 8D). This test of creep deformation and recovery provides insights into how well a filler maintains its shape after enduring continuous deformation, due to the inherent cohesion that aids in recovery once the deforming force is removed.

From these experimental results, it can be concluded that biphasic HA fillers, with their fundamentally rigid particle structure, are more capable of forming and maintaining large structures and exhibit better elasticity to withstand external forces. However, it is not necessarily the case that larger particles in monophasic HA fillers will always perform better under external force. Although these fillers are similar in viscoelastic properties, larger particles create greater spacing between them, leading to increased deformation rates and more severe deformations under external forces. The cohesion among closely packed particles is generally stronger in fillers with smaller particles compared to those with larger ones. Nonetheless, when not subjected to external forces, larger-particle fillers may create a smoother overall volume, potentially making them more suitable for specific facial areas depending on skin and tissue density, the extent of external force, and the area’s mobility. Therefore, the choice of which monophasic filler to use should be guided by these considerations [22,24,25,28,29].

### 4.6. Flexibility Test

The flexibility test evaluates how well filler particles retain their original form when subjected to stretching stimuli, either horizontally or vertically, and assesses their ability to adapt and return to their initial structure after being elongated or compressed.

**Figure 9:**
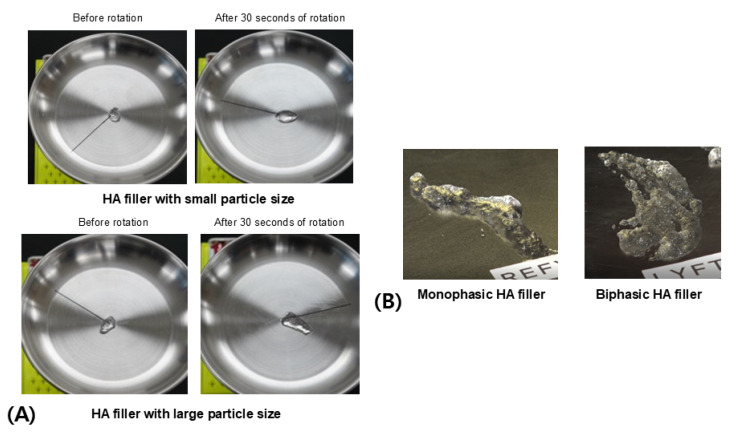
Comparison of response to centrifugal force by rotation between HA fillers with different particle sizes (**A**). Difference in flexibility between monophasic and biphasic HA fillers (**B**). The figures are produced by the authors.

Biphasic HA fillers, characterized by HA molecules that naturally entangle like yarn balls and resist easy unraveling, inherently exhibit less flexibility due to their tightly interwoven structural composition. These fillers generally demonstrate limited extension when spread laterally and are prone to breaking midway. In contrast, monophasic HA fillers, with HA molecules arranged in a simpler, less tangled configuration, display greater flexibility. These fillers can stretch further without breaking, forming longer, uninterrupted strands when pulled laterally (Figure 9A).

An important consideration is whether the final monophasic HA filler product contains free HA, as this significantly impacts the product’s flexibility [9,30,31]. While fillers with similar viscoelasticity indices that include free HA may feel softer and more fluid to the touch—similar to the effect of water absorption—their structural integrity is often compromised. The presence of water molecules interspersed among the HA chains weakens the structural cohesion, making these fillers more susceptible to breaking under lateral stress. Consequently, fillers that do not contain free HA tend to better recover their original form and exhibit greater durability under dynamic conditions (Figure 9B) [1].

Thus, when selecting fillers for areas with frequent movement, such as around the mouth, it is essential not only to consider the filler’s softness but also to verify whether the product contains free HA. This ensures that the chosen filler can adequately withstand and recover from the natural movements of the face, thereby providing both desirable results and lasting durability in highly mobile or expressive areas.

## 5. Discussion

The review of the rheological characteristics of HA fillers highlights the importance of understanding the viscoelastic properties of these materials for their effective application in clinical settings. HA fillers are categorized into biphasic and monophasic types, each exhibiting distinct viscoelastic behaviors due to their unique manufacturing processes [1,32]. Biphasic fillers, characterized by minimal BDDE cross-linking, rely on natural entanglement for their structural integrity. This results in lower degrees of chemical modification (MoD) and generally lower modification efficiency (MoE). In contrast, monophasic fillers undergo extensive chemical cross-linking, leading to higher MoD values, which contribute to a firmer texture and enhanced resistance to enzymatic degradation [32,33,34,35,36].

The study by Fundaro et al. [12] reviewed various HA fillers used for facial volume augmentation and wrinkle correction, noting key rheological properties such as storage modulus (G′), loss modulus (G″), complex modulus (G*), tangent delta (tan δ), cohesivity, and complex viscosity (η*). These properties influence the fillers’ elastic and viscous behavior, injection ease, and tissue integration. For example, Juvederm Ultra 3 has a G′ of 173.28 ± 20.63 Pa and a viscosity of 1629.90 ± 233.33 Pas, making it suitable for treating wrinkles between the nose and mouth. In comparison, Juvederm Ultra 4, with a G′ of 102.21 ± 11.46 Pa and a viscosity of 1479.10 ± 75.41 Pas, is more appropriate for severe folds and facial contouring. Juvederm Voluma, designed for volume loss in the cheeks, has a G′ of 603.14 ± 58.34 Pa and a viscosity of 1033.40 ± 50.37 Pas. Belotero Soft, used for fine superficial folds like crow’s feet, has a G′ of 6.93 ± 0.73 Pa and a viscosity of 149.09 ± 46.19 Pas, while Belotero Intense, intended for deep folds and lip volume augmentation, has a G′ of 76.41 ± 7.90 Pa and a viscosity of 1008.70 ± 115.06 Pas. Restylane, suitable for creases, wrinkles, scars, and lip enhancement, has a G′ of 301.08 ± 8.55 Pa and a viscosity of 230.35 ± 61.25 Pas. These values align with our findings, showing no significant differences.

Choi [37] observed that Restylane, a biphasic filler, varies in particle size, whereas Juvéderm, a monophasic filler, is controlled by the degree of cross-linking. Understanding the rheological properties of these fillers—such as viscoelasticity (G*, G′, and G″), tan δ, and cohesivity—is crucial for optimizing clinical outcomes. Viscoelasticity describes the material’s viscosity and elasticity, affecting how fillers deform and restore their shape under shear forces. Cohesivity measures internal adhesion among HA units, influencing the filler’s behavior under compression. Different facial areas require fillers with specific properties: high G′ for volumizing and low G′ for fine wrinkles. High G′ fillers are ideal for volumizing the forehead and nasolabial folds, while low G′ fillers are better suited for fine wrinkle correction.

Guardia et al. [38] summarized rheological data on HA fillers provided by manufacturers (Table 2). The data presented in this study are consistent with those reported by Guardia et al., showing no significant differences.

Understanding histological changes in tissue response to HA fillers is essential for optimizing clinical outcomes. The formation of collagen capsules and the gradual replacement of filler material with autologous tissue underscore the dynamic integration process of HA fillers [19,36]. Clinically, this knowledge assists practitioners in selecting appropriate fillers based on injection sites, patient-specific tissue conditions, and desired outcomes [30]. The review emphasizes the need for fillers to balance viscoelastic properties with safety, ensuring that they can maintain their shape under external pressures while minimizing adverse reactions. This balanced approach allows for the selection of fillers that provide both immediate esthetic benefits and long-term patient satisfaction.

Biphasic and monophasic HA fillers exhibit distinct rheological properties that significantly affect their clinical performance and safety profiles. Biphasic fillers generally demonstrate higher storage moduli, offering greater structural support and longevity. In contrast, monophasic fillers are often more cohesive and better suited for areas requiring smoother integration with surrounding tissues. Achieving a balance between viscoelastic properties and safety is crucial, with an optimal storage modulus range identified to achieve desired esthetic outcomes while minimizing risks. This research highlights the importance of understanding the rheological characteristics of HA fillers to guide their appropriate clinical application and enhance patient satisfaction.

## Figures and Tables

**Table 1:** This table summarizes the factors influencing HA filler cohesion based on these clinical phenomena. This table is produced by the authors.

Factors Related to the Cohesion of Hyaluronic Acid Fillers
Concentration of hyaluronic acid
2. Molecular weight of hyaluronic acid
3. Type and degree of cross-linking of hyaluronic acid filler
4. Degree and consistency of particle size of hyaluronic acid filler
5. Enough hydration during the stirring stage
6. Stable hydrogen bond
7. Fluidity of hyaluronic acid filler (phase angle value)

**Table 2:** Summary of hyaluronic acid concentration, viscoelastic properties, tan delta values, cohesivity, and maximum water uptake for various HA fillers. This table is produced by the authors.

Filler Product Name	HA (mg/mL)	G′ 5 Hz (Pa)	G″ 5 Hz (Pa)	Tan d	Cohesivity/Fn (gmf)	Maximum Water Uptake (%)
Restylane Fynesse	20	134	58	0.433	30	677
Restylane Refyne	20	116	50	0.431	49	516
Restylane Kysse	20	236	50	0.212	85	373
Restylane Defyne	20	342	47	0.137	60	318
Restylane Volyme	20	239	50	0.209	91	354
Restylane Vital Light	12	84	49	0.583	12	<100
Restylane Vital	20	667	172	0.258	27	<100
Restylane	20	864	185	0.214	29	<100
Restylane Lyps	20	976	166	0.170	31	<100
Restylane Lyft	20	977	198	0.203	32	<100
Restylane SubQ	20	1055	123	0.117	42	<100
Juvéderm Ultra	24	156	68	0.436	96	580
Juvéderm Ultra XC	24	207	80	0.386	96	622
Juvéderm Ultra Plus	24	214	74	0.346	116	515
Juvéderm Ultra Plus XC	24	263	79	0.300	112	454
Juvéderm Ultra 2	24	188	75	0.399	95	574
Juvéderm Ultra 3/Smile	24	238	71	0.298	104	426
Juvéderm Ultra 4	24	164	66	0.402	105	614
Juvéderm Volite	12	166	30	0.181	12	<100
Juvéderm Volbella with lidocaine	15	271	39	0.144	19	133
Juvéderm Volift with lidocaine	17.5	340	46	0.135	30	184
Juvéderm Voluma with lidocaine	20	398	41	0.103	40	227
Juvéderm Volux	25	665	49	0.074	93	253
Teosyal Puresense Redensity II	15	114	43	0.372	16	239
Teosyal Puresense First Lines	20	105	44	0.419	18	250
Teosyal Puresense Kiss	25	314	66	0.209	74	380
Teosyal Puresense Deep Lines	25	301	64	0.214	82	300
Teosyal Puresense Ultra Deep	25	348	54	0.155	87	250
Teosyal RHA1	15	133	54	0.406	22	260
Teosyal RHA2	23	319	99	0.310	77	420
Teosyal RHA3	23	264	67	0.254	109	427
Teosyal RHA4	23	346	62	0.179	115	366
Belotero Soft	20	40	42	1.050	16	<100
Belotero Balance/Lips Contour	22.5	128	82	0.641	69	664
Belotero Intense/Lips Shape	25.5	255	110	0.431	115	700
Belotero Volume	26	438	103	0.235	97	370

## References

[1] Broder K.W., Cohen S.R. (2006). An overview of permanent and semipermanent fillers. Plast. Reconstr. Surg..

[2] Stern R. (2004). Hyaluronan catabolism: A new metabolic pathway. Eur. J. Cell Biol..

[3] Woodward J., Khan T., Martin J. (2015). Facial Filler Complications. Facial Plast. Surg. Clin. N. Am..

[4] Fraser J.R., Laurent T.C. (1989). Turnover and metabolism of hyaluronan. Ciba Found. Symp..

[5] Lemperle G., Rullan P.P., Gauthier-Hazan N. (2006). Avoiding and treating dermal filler complications. Plast. Reconstr. Surg..

[6] Nettar K., Maas C. (2012). Facial filler and neurotoxin complications. Facial Plast. Surg..

[7] Chang S.H., Yousefi S., Qin J., Tarbet K., Dziennis S., Wang R., Chappell M.C. (2016). External Compression Versus Intravascular Injection: A Mechanistic Animal Model of Filler-Induced Tissue Ischemia. Ophthalmic Plast. Reconstr. Surg..

[8] DeLorenzi C. (2017). New High Dose Pulsed Hyaluronidase Protocol for Hyaluronic Acid Filler Vascular Adverse Events. Aesthet. Surg. J..

[9] Liu M.H., Beynet D.P., Gharavi N.M. (2019). Overview of Deep Dermal Fillers. Facial Plast. Surg..

[10] DeLorenzi C. (2013). Complications of injectable fillers, part I. Aesthet. Surg. J..

[11] Huang Y., Zhang Y., Fei X., Fan Q., Mao J. (2022). Monophasic and Biphasic Hyaluronic Acid Fillers for Esthetic Correction of Nasolabial Folds: A Meta-Analysis of Randomized Controlled Trials. Aesthetic Plast. Surg..

[12] Fundarò S.P., Salti G., Malgapo D.M.H., Innocenti S. (2022). The Rheology and Physicochemical Characteristics of Hyaluronic Acid Fillers: Their Clinical Implications. Int. J. Mol. Sci..

[13] Chun C., Lee D.Y., Kim J.T., Kwon M.K., Kim Y.Z., Kim S.S. (2016). Effect of molecular weight of hyaluronic acid (HA) on viscoelasticity and particle texturing feel of HA dermal biphasic fillers. Biomater. Res..

[14] Sundaram H., Voigts B., Beer K., Meland M. (2010). Comparison of the rheological properties of viscosity and elasticity in two categories of soft tissue fillers: Calcium hydroxylapatite and hyaluronic acid. Dermatol. Surg..

[15] De Boulle K., Glogau R., Kono T., Nathan M., Tezel A., Roca-Martinez J.X., Paliwal S., Stroumpoulis D. (2013). A review of the metabolism of 1,4-butanediol diglycidyl ether-crosslinked hyaluronic acid dermal fillers. Dermatol. Surg..

[16] Borrell M., Leslie D.B., Tezel A. (2011). Lift capabilities of hyaluronic acid fillers. J. Cosmet. Laser Ther..

[17] Hong G.-W., Yi K.-H. (2024). Skin & SMAS layer remodeling technique (SSRT): Achieving both volume and lifting effects with filler treatments. Skin Res. Technol..

[18] Kim H.J., Kwon S.B., Whang K.U., Lee J.S., Park Y.L., Lee S.Y. (2018). The duration of hyaluronidase and optimal timing of hyaluronic acid (HA) filler reinjection after hyaluronidase injection. J. Cosmet. Laser Ther..

[19] Ozturk C.N., Li Y., Tung R., Parker L., Piliang M.P., Zins J.E. (2013). Complications following injection of soft-tissue fillers. Aesthet. Surg. J..

[20] La Gatta A., De Rosa M., Frezza M.A., Catalano C., Meloni M., Schiraldi C. (2016). Biophysical and biological characterization of a new line of hyaluronan-based dermal fillers: A scientific rationale to specific clinical indications. Mater. Sci. Eng. C Mater. Biol. Appl..

[21] Christensen L., Breiting V., Janssen M., Vuust J., Hogdall E. (2005). Adverse reactions to injectable soft tissue permanent fillers. Aesthetic Plast. Surg..

[22] Perera G.G.G., Argenta D.F., Caon T. (2024). The rheology of injectable hyaluronic acid hydrogels used as facial fillers: A review. Int. J. Biol. Macromol..

[23] Casabona G. (2015). Blood Aspiration Test for Cosmetic Fillers to Prevent Accidental Intravascular Injection in the Face. Dermatol. Surg..

[24] Budai L., Budai M., Fülöpné Pápay Z.E., Vilimi Z., Antal I. (2023). Rheological Considerations of Pharmaceutical Formulations: Focus on Viscoelasticity. Gels.

[25] Zhou W., Hou S., Deng S., Peng Y., Fu W., Zhou Y., Yang J., Peng C. (2023). The Intrinsic Relation between the Hydrogel Structure and In Vivo Performance of Hyaluronic Acid Dermal Fillers: A Comparative Study of Four Typical Dermal Fillers. Tissue Eng. Regen. Med..

[26] Wongprasert P., Dreiss C.A., Murray G. (2022). Evaluating hyaluronic acid dermal fillers: A critique of current characterization methods. Dermatol. Ther..

[27] Kim K.T., Lee W., Yang E.J. (2024). “Cohesiveness of Hyaluronic Acid Fillers”: Evaluation Using Multiple Cohesion Tests. Arch. Plast. Surg..

[28] Edsman K.L., Wiebensjö Å.M., Risberg A.M., Öhrlund J. (2015). Is There a Method That Can Measure Cohesivity? Cohesion by Sensory Evaluation Compared With Other Test Methods. Dermatol. Surg..

[29] Zerbinati N., Capillo M.C., Sommatis S., Maccario C., Alonci G., Rauso R., Galadari H., Guida S., Mocchi R. (2022). Rheological Investigation as Tool to Assess Physicochemical Stability of a Hyaluronic Acid Dermal Filler Cross-Linked with Polyethylene Glycol Diglycidyl Ether and Containing Calcium Hydroxyapatite, Glycine and L-Proline. Gels.

[30] Sundaram H., Signorini M., Liew S., Trindade de Almeida A.R., Wu Y., Vieira Braz A., Fagien S., Goodman G.J., Monheit G., Raspaldo H. (2016). Global Aesthetics Consensus: Botulinum Toxin Type A--Evidence-Based Review, Emerging Concepts, and Consensus Recommendations for Aesthetic Use, Including Updates on Complications. Plast. Reconstr. Surg..

[31] Signorini M., Liew S., Sundaram H., De Boulle K.L., Goodman G.J., Monheit G., Wu Y., Trindade de Almeida A.R., Swift A., Vieira Braz A. (2016). Global Aesthetics Consensus: Avoidance and Management of Complications from Hyaluronic Acid Fillers-Evidence- and Opinion-Based Review and Consensus Recommendations. Plast. Reconstr. Surg..

[32] Alijotas-Reig J., Garcia-Gimenez V. (2008). Delayed immune-mediated adverse effects related to hyaluronic acid and acrylic hydrogel dermal fillers: Clinical findings, long-term follow-up and review of the literature. J. Eur. Acad. Dermatol. Venereol..

[33] Curi M.M., Cardoso C.L., Curra C., Koga D., Benini M.B. (2015). Late-onset adverse reactions related to hyaluronic Acid dermal filler for aesthetic soft tissue augmentation. J. Craniofacial Surg..

[34] Beleznay K., Carruthers J.D., Carruthers A., Mummert M.E., Humphrey S. (2015). Delayed-onset nodules secondary to a smooth cohesive 20 mg/mL hyaluronic acid filler: Cause and management. Dermatol. Surg..

[35] Sorensen E.P., Urman C. (2015). Cosmetic complications: Rare and serious events following botulinum toxin and soft tissue filler administration. J. Drugs Dermatol..

[36] Alhede M., Er Ö., Eickhardt S., Kragh K., Alhede M., Christensen L.D., Poulsen S.S., Givskov M., Christensen L.H., Høiby N. (2014). Bacterial biofilm formation and treatment in soft tissue fillers. Pathog. Dis..

[37] Choi M.S. (2020). Basic rheology of dermal filler. Arch. Plast. Surg..

[38] de la Guardia C., Virno A., Musumeci M., Bernardin A., Silberberg M.B. (2022). Rheologic and Physicochemical Characteristics of Hyaluronic Acid Fillers: Overview and Relationship to Product Performance. Facial Plast. Surg..

